# Impact of adverse events of antiretroviral treatment on regimen change and mortality in Ugandan children

**DOI:** 10.4102/phcfm.v2i1.109

**Published:** 2010-06-04

**Authors:** Ntambwe Malangu, Yvonne Karamagi

**Affiliations:** 1 Department of Epidemiology, University of Limpopo, South Africa

**Keywords:** Antiretroviral, treatment, adverse, events, children

## Abstract

**Background:**

Outcomes of antiretroviral treatment have been documented in both developed and developing countries. It has been reported consistently that the treatment is associated with many adverse events. However, little is known about their impact on the quality of life, clinical management, and survival in children aged less than 6 years in Uganda.

**Objectives:**

The purpose of this study was to determine the prevalence of the adverse events of antiretroviral treatment, their impact on mortality and the change in regimens prescribed to children treated at Mildway Centre in Uganda.

**Method:**

A retrospective chart review was performed for children younger than 6 years, treated since the Mildway Centre was opened in 1999. In order to achieve a larger sample, the records of children treated from January 2000 to July 2005 were included in the study. A pre-tested data collection form was used to collate socio-demographic and clinical data of the patients. These included the documented adverse events, causes of death, stage of infection, duration of treatment, regimen prescribed, year of enrolment into the treatment program, as well as whether or not they were still alive. Descriptive statistics were used in the analysis of data.

**Results:**

Of the 179 children, the majority were males and had a median age of 4 years. The majority (58.8%) of children had suffered from severe immune depression since they met the WHO clinical stage III and IV, 73.8% had a baseline CD4T of less than 15%. Four regimens were prescribed to the children. The most common was a regimen containing zidovudine, lamivudine, and nevirapine (34.6%), followed by a regimen containing stavudine, lamivudine, and nevirapine (27.9%). Eleven children (6.1%) had their regimen changed, of which six (54.5%) were due to adverse events. The prevalence of adverse events was 8%; of the 14 documented adverse events, the most common were severe anaemia (3), vomiting (3), and skin rashes (3). After 12 months on treatment, 8% of the patients had died. The most common causes of death were infectious diseases (28.6%), severe anaemia (21.4%), and severe dehydration (21.4%).

**Conclusion:**

The prevalence of adverse events was 8%; they were responsible for 54.5% of regimen changes and 21.4% of deaths in children treated at the study site. These findings suggest the need for incorporating pharmacovigilance practices into the provision of antiretroviral treatment.

## INTRODUCTION

Outcomes of antiretroviral treatment have been documented in both developed and developing countries. It has been reported consistently that the treatment is associated with many adverse events including gastro-intestinal disturbances, peripheral neuropathy and others.^1–3^ These adverse events impact not only the quality of life of the patients but also their clinical management and survial.^4, 5^ However, little is known about these processes in children aged less than 6 years treated at the study site. Therefore, the purpose of this study was to determine the prevalence of the adverse events of antiretroviral treatment and their impact on the mortality and change in regimens prescribed to children treated at Mildway Centre in Uganda.

## METHOD

A retrospective chart review was performed for children aged less than six years treated since the centre opened in 1999. In order to achieve a larger sample, the records of children treated from January 2000 to July 2005 were included in the study. Age at the time of enrolment was used for inclusion in the study. Besides age, the other inclusion criteria were that the children should have been on treatment for at least 12 months, with the treatment having been initiated at Mildway Centre. Based on the treatment registers, 202 children had been treated during this period. Of these, 16 records could not be retrieved, while seven records did not meet one of the inclusion criteria in that these children had not completed at least 12 months on antiretroviral treatment. Hence, 179 records were included in the final analysis. A pre-tested data collection form was used to collate the socio-demographic and clinical data of the patients. These included the documented adverse events, causes of death, stage of infection, duration of treatment, regimen prescribed, year of enrolment into the treatment program, as well as whether or not they were still alive. Descriptive statistics were calculated but no statistical testing was conducted. All statistical analyses were performed using SPSS software (version 17.0, SPSS, Chicago, IL, USA). The approval to conduct this study was obtained from the Medunsa Campus Research and Ethics Committee of the University of Limpopo; while the permission to access the patients’ records was requested and obtained from the management of Mildway Centre.

## RESULTS

**FIGURE 1:**
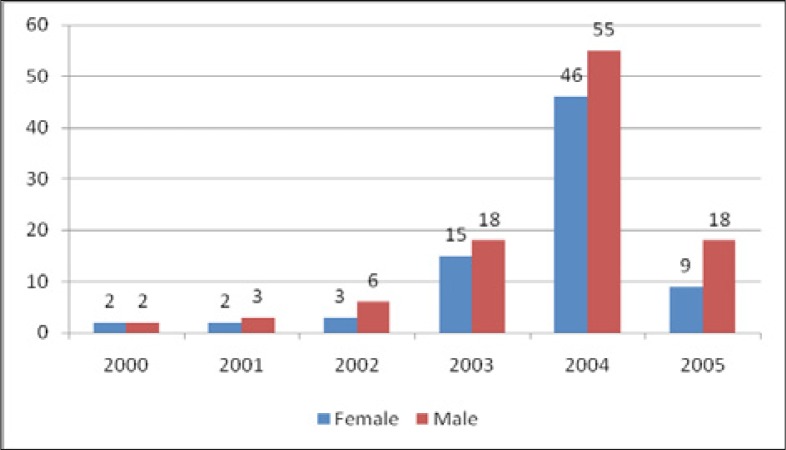
Enrolment of children into antiretroviral program by sex

As shown in Table 1, of the 179 children, 53.1% were 4–5 years old. Their median age was four years, or 48 months. Their age ranged from 4 to 71 months. About 57% of the children enrolled for treatment were males. The majority (58.8%) of children had suffered from severe immune depression since they met the WHO clinical stage III and IV; 73.8% had a baseline CD4T of less than 15%. All children had been prescribed cotrimoxazole as a prophylaxis for opportunistic infections. Four regimens were prescribed to the children; the most common was a regimen containing zidovudine, lamivudine and nevirapine. The enrolment into the antiretroviral treatment program for children started in 2000, though the majority of patients were enrolled in 2004 (Figure 1). The mean duration of treatment was 13.4 months, only 7.3% of children had been on treatment for more than a year.

**TABLE 1:** Characteristics of children included in the study

Variables	Frequency	%
**Sex (*n* = 179)**		
Female	76	43
Male	102	57
**Age in categories (*n* = 179)**		
< 1 year (0-11 months)	3	1.7
1 – < 2 years (12-23 months)	13	7.3
2 – < 3 years (24-35 months)	39	21.8
3 – < 4 years (36-47 months)	29	16.2
4 – < 5 years (48-59 months)	55	30.7
5 – < 6 years (60-71 months)	40	22.3
**WHO HIV Clinical Stage (*n* = 114)**		
Stage I and II	47	41.2
Stage III and IV	67	58.8
**Baseline CD4T per cent (*n* = 149)**		
CD4% ≤ 15% (severe immune suppression)	110	73.8
CD4% > 15%	39	26.2
**Regimen prescribed (*n* = 179)**		
Zidovudine + Lamivudine + Nevirapine	62	34.8
Stavudine + Lamivudine + Nevirapine	50	28.1
Zidovudine + Lamivudine + Efavirenz	43	24.2
Stavudine + Lamivudine + Efavirenz	23	12.9

Eleven children (6.1%) had their regimen changed. The majority of regimen changes occurred during the first six months of treatment. The reasons for changing the regimen were due to adverse events in six cases (54.5%), occurrence of opportunistic infections such as tuberculosis and pneumonia (18.2%), poor adherence (18.2%) and in one case (9.1%) the reason was not recorded. The adverse events involved in regimen changes were severe anaemia (three cases), skin rashes (two cases), and peripheral neuropathy (one case).

The prevalence of adverse events was 8%. The most common were anaemia, vomiting and skin rashes. Other adverse events included diarrhoea, immune reconstruction syndrome, jaundice and peripheral neuropathy. These adverse events impacted substantially on the children's treatment; in six children the regimen had to be changed, while in two other children the treatment had to be stopped (Table 2).

**TABLE 2:** Types of adverse events and impacts in children

Variables	Frequency	%
**Types of adverse events**		
Nausea and vomiting	3	21.4
Skin rashes	3	21.4
Severe anaemia	3	21.4
Jaundice	2	14.3
Peripheral neuropathy	1	7.1
Immune reconstitution syndrome	1	7.1
Diarrhoea	1	7.1
**Type of impact on patients’ regimens**		
Patients’ ART regimen was changed	6	42.9
Patients’ ART regimen was stopped	3	21.4
Patients’ ART regimen remained unchanged	3	21.4
Patient was put on drug holiday	2	14.3

**FIGURE 2:**
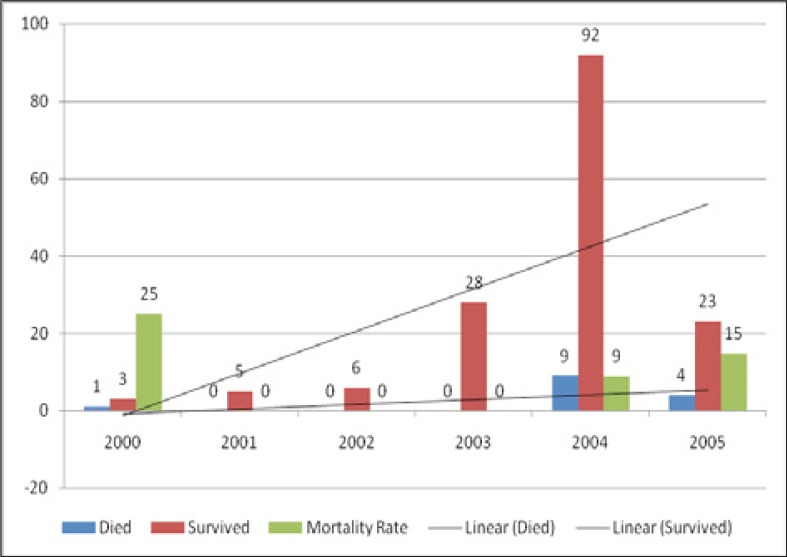
Mortality rate per year

At the end of the first year on antiretroviral treatment, the mortality rate was 8%. Three deaths occurred within the first month, while six others followed within two to six months of treatment. Of the 14 children who died, the most common causes of death were: infectious diseases (28.6%) such as chicken pox, malaria, pneumonia, and tuberculosis, adverse events such as severe anaemia (21.4%), and severe dehydration (21.4%). One death (7.1%) was due to severe malnutrition, while the cause of death was not specified in three cases (21.4%). The characteristics of those who died were as follows: all but one was younger than 2 years old, 10 were female, and 11 had a baseline CD4T of less than 15%, an indication of severe immunosuppression. Moreover, while one of the four children enrolled in 2000 died (25%), no child enrolled in 2001, 2002, and 2003, had died. Most of the deaths occurred in children enrolled in 2004 and 2005, nine out of 101 (9%), and four out of 27 (15%), respectively (Figure 2).

## DISCUSSION

Access to antiretroviral treatment by children is limited in Uganda. Although the Mildway Centre has been operational since 1999, it was the advent of international programs such as the President's Plan for AIDS Relief and the Global Fund for HIV, Malaria and Tuberculosis that facilitated the increase in the scale-up of antiretroviral treatment for Ugandan children. This explains why the number of children on antiretroviral treatment increased substantially from 2004 onwards. However, it is still unclear why more male than female children were enrolled (Figure 1).

With regard to the prevalence of adverse events, it was 8%. This figure is well within the range of between 2.5% – 29% as reported in the literature.^7–9^ This finding clearly demonstrates a good tolerance of antiretroviral treatment in this group of children. This has been reported in other settings.^1, 3, 6, 9, 10^ In this study, the range of adverse events documented was limited. In fact, only seven adverse events were documented within the first 6 months of treatment among 14 patients. The most common were anaemia, vomiting and skin rashes. Apart from anaemia, for which some indication of severity was indicated, there was no such information on other adverse events.

The profile of adverse events is consistent with previous studies at the same facility, and reports from studies about antiretroviral treatment in children in other African countries.^11–13^ Moreover, in contrast to other studies, this study has established that adverse events were responsible for over half of regimen changes effected among the sample, as well as the institution of a drug holiday for two children and the cessation of the treatment in three other cases. This substantial impact suggests that children on antiretroviral treatment should be monitored using pharmacovigilance skills and tools. During the period covered by this study, Uganda did not have any national pharmacovigilance centre and to date none exists. There is a need for such a centre.

With regard to regimens changed, it is noteworthy that all four consisted of two nucleoside reverse-transcriptase inhibitors (lamivudine with either, zidovudine or stavudine) plus one non-nucleoside reverse-transcriptase efavirenz or nevirapine). The second-line drugs such as didanosine and abacavir, and protease inhibitors such as lopinavir-ritonavir or nelfinavir were not prescribed even though some children were supposedly on rifampicin as they suffered from tuberculosis. The regimen change of 6.1% reported here is somewhat lower as compared to reports by other investigators.^4, 14^

With regard to mortality, the rate of 8% reported in this study is consistent with previous findings in children in other settings in Africa. However, on a yearly basis, the mortality rate varied from 0% – 25%. In a multi-centre study involving nine countries, Carter et al.^15^ reported a mortality of 9%. These findings concur with a well-demonstrated fact that antiretroviral treatment does save lives, particularly in children because without it, it is estimated that 80% of HIV-Infected children would die before their fifth birthday.^16^

Of the 14 deaths reported in this study, infectious diseases were the most common cause of death followed by severe dehydration, and adverse events. The adverse event associated with death was severe anaemia – a typical side effect for regimens containing zidovudine. This finding emphasises again the need for close monitoring involving both clinical and biological parameters so that appropriate interventions could be implemented without unnecessary delays. In this study, over half of the deaths occurred within the first six months of treatment; 11 of 14 deaths occurred in children who had a CD4T of less than 15%. This suggests that these children were brought for treatment when their immune systems were already severely compromised. This finding concurs with the view that the late initiation of antiretroviral treatment is associated with poor outcomes.^12, 13, 17, 18^ In order to improve treatment outcomes, healthcare providers of antiretroviral therapy to children must be aware of the causes of mortality and the complications of antiretroviral treatment so that they can act swiftly to increase the chance of survival for these children. Interventions, such as ensuring adherence to antiretroviral treatment guidelines, adherence to prescribed treatment by patients, as well as the identification of adverse events and treating them appropriately will contribute to effective care. In order to implement the last intervention, a national drive at policy-making level is required to instil the concept and practice of pharmacovigilance in clinical practice.

Finally, given the design of this study, causal relationships could not be established, nor the reasons for the disproportionate enrolment of male children and the deaths of female patients. Other limitations include the possibility that some adverse events may have not been recorded or have been misinterpreted by the healthcare providers.

## CONCLUSION

The prevalence of adverse events was 8%, which was responsible for 54.5% of regimen change, and 21.4% deaths in children treated at the study site. These findings suggest the need for incorporating pharmacovigilant practice into the provision of antiretroviral treatment.
